# P-1318. Epidemiology and Trends of *Corynebacterium diphtheriae* in the United States, 2016-2023

**DOI:** 10.1093/ofid/ofae631.1498

**Published:** 2025-01-29

**Authors:** Isha Berry, Valerie Bampoe, Matthew Cole, Hong Ju, Ingrid C McCall, Marissa Fraire, Heather Fueston, Lucia Pawloski, Farrell A Tobolowsky, Erin Tromble

**Affiliations:** CDC, Atlanta, Georgia; CDC - Centers for Disease Control and Prevention, Atlanta, Georgia; CDC/NCIRD/DBD/MVPDB, Atlanta, Georgia; CENTERS FOR DISEASE CONTROL ATLANTA, Atlanta, Georgia; CENTERS FOR DISEASE CONTROL AND PREVENTION (CDC), Atlanta, Georgia; Centers for Disease Control & Prevention, Atlanta, Georgia; Centers for Disease Control and Prevention, Atlanta, Georgia; Centers for Disease Control and Prevention, Atlanta, Georgia; CDC, Atlanta, Georgia; CDC, Atlanta, Georgia

## Abstract

**Background:**

Diphtheria, commonly resulting in severe respiratory or cutaneous disease, is caused by toxin-producing (i.e., toxigenic) strains of *Corynebacterium diphtheriae*. Non-toxigenic *C. diphtheriae* also cause disease, which is typically less severe but can be invasive. Non-toxigenic bacteria can acquire and express the toxin (*tox)* gene, providing a mechanism for diphtheria re-introduction to the United States (US). We examine the epidemiology, toxigenicity, and trends of *C. diphtheriae* isolates submitted to CDC from January 2016 to September 2023.

Corynebacterium Diphtheriae Isolates in the United States, by year

**Figure:**
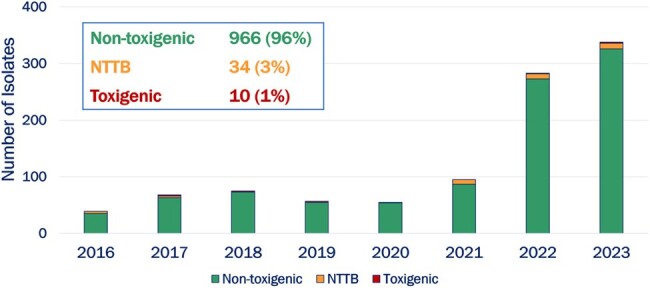

**Methods:**

*C. diphtheriae* isolates submitted to CDC were cultured and tested by polymerase chain reaction for species confirmation and *tox* detection; toxin production was confirmed by Elek. Laboratory data were linked with epidemiological case data. Isolates were classified as non-toxigenic if they were *tox-*negative or toxigenic if *tox-*positive and Elek-positive. We examined temporal trends in reported isolates and describe demographic and epidemiologic case characteristics.

**Results:**

A total of 1010 *C. diphtheriae* isolates were identified during 2016—2023; 99.0% (n=1000) were non-toxigenic, and 1.0% (n=10) were toxigenic. An annual average of 65 (range: 39-95) isolates were reported during 2016—2021; in 2022 and 2023 this increased to an average of 311 (range: 283-338) isolates reported per year. Median patient age was 43 years (range: < 1-98), and 69.0% (n=697) were male. Isolates were primarily obtained from cutaneous (n=689, 68.2%), respiratory (n=133, 13.2%) and blood (n=127, 12.6%) sources. Among non-toxigenic cases with epidemiological data (n=800), major risk factors included housing instability (28.2%) and history of IVDU (20.9%). Eight of the 10 toxigenic cases reported recent international travel to a diphtheria-endemic country.

**Conclusion:**

There has been an increase in *C. diphtheriae* isolates identified in the US; however, the majority of isolates were non-toxigenic and from cutaneous sites. Though toxigenic cases were infrequent, risk factors appear to differ between toxigenic compared with non-toxigenic cases. Awareness of case risk factors may help to effectively guide public health intervention and infection prevention and control measures prior to toxigenicity confirmation.

**Disclosures:**

**All Authors**: No reported disclosures

